# Supplementary material for: Adverse Maternal Environments Perturb Hepatic DNA Methylome and Transcriptome Prior to the Adult-Onset Non-Alcoholic Fatty Liver Disease in Mouse Offspring
Source: Nutrients. 2023 Apr 30;15(9):2167. doi: 10.3390/nu15092167 (PMC10180730; doi:10.3390/nu15092167)
Supplement: Supplementary file 1 [file nutrients-15-02167-s001.zip › Supplementary Table S1 and Figure S1-S3.pdf]

Supplementary Table S1 Primers/probe information for real-time RT-PCR

Genes	Primers/probe catalog number	Reference sequence
Il1b	Mm.PT.58.41616450[1]	NM_008361.1
Ces1g	Mm.PT.58.10501081[1]	NM_021456.1
Lpin1	Mm.PT.58.6349353[1]	NM_001130412.3
Elovl3	Mm.PT.58.5393956[1]	NM_007703.1
Acaca	Mm01304257_m1[2]	NM_133360.2
Hprt	Mm.PT.39a.22214828[1]	NM_013556.2

[1]Integrated DNA Technologies; [2]ThermoFisher Scientific.

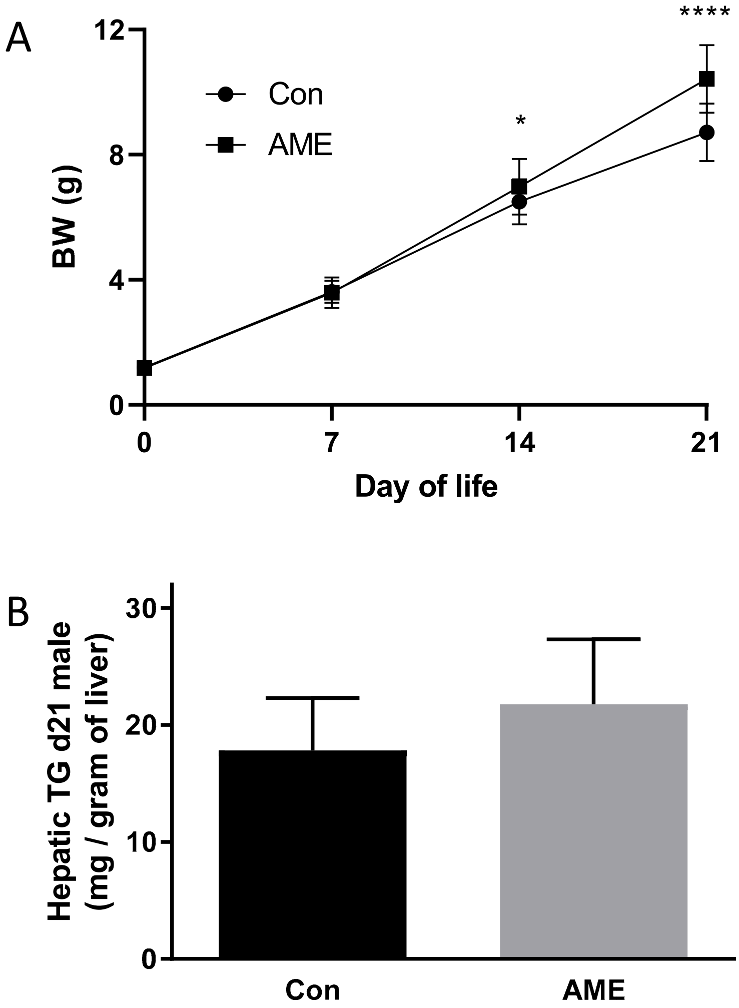

Supplementary Figure S1. Effect of AME on bodyweight (A) and hepatic TG contents (B) in offspring. (A) the changes of bodyweight over time from d0 to d21 in male mouse offspring. (B) The hepatic TG contents at d21 in male mouse livers. Values are means ± SDs. * $p<0.05$; **** $p<0.0001$. AME, adverse maternal environment; BW, bodyweight; Con, control; TG, triglyceride.

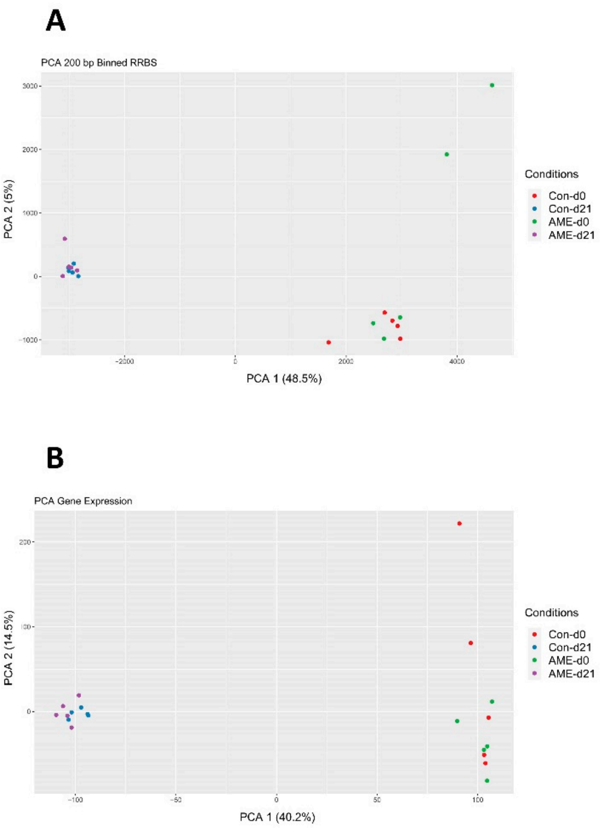

Supplementary Figure S2. **PCA plots for DNA methylation of CpG sites (A) and gene expression (B)**. AME, Adverse maternal environment; Con, control; PCA, Principal component analysis.

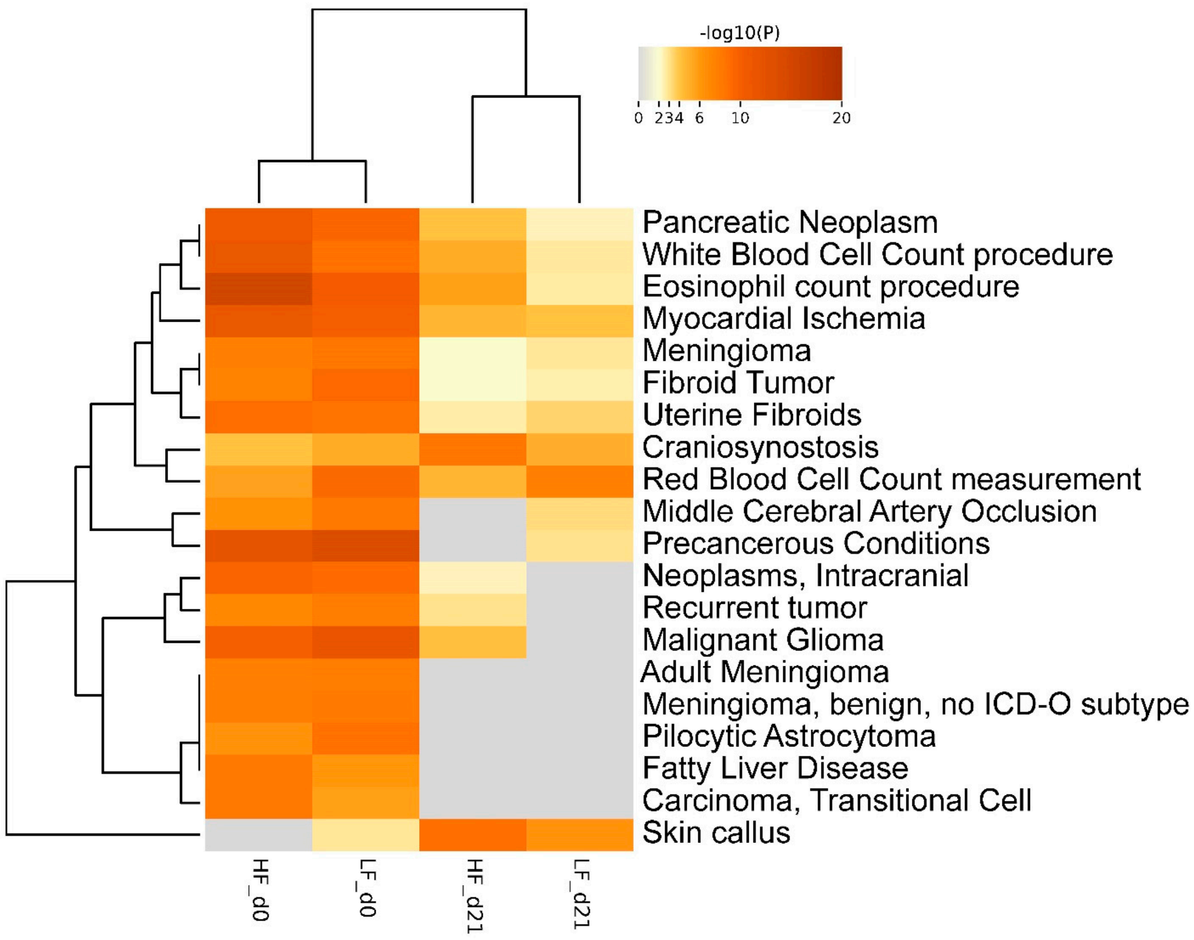

Supplementary Figure S3. **Disease heatmap based on genes linked to distal elements overlapped with AME-resulted DMRs, which were analyzed against publicly available Promoter Capture Hi-C dataset**. d0 and d21 AME-resulted DMR coordinates were converted to mm9 using CrossMap [1], which were then intersected against the provided end coordinates of HF (n= 151203) and LF (n= 149798) cis Promoter Capture Hi-C interactions from the publicly available study GSE124463 [2]. We extracted the genes for the RefSeq gene TSS provided by the study for both end1 and end2 of each overlapped interaction. DisGeNET enrichment analysis [3] was used on each list of genes to identify overrepresented UMLS diseases. HF, high lipid diet; LF, high carbohydrate diet; UMLS, unified medical language system.

Reference

1. Zhao H, Sun Z, Wang J, Huang H, Kocher JP and Wang L (2014) CrossMap: a versatile tool for coordinate conversion between genome assemblies. Bioinformatics 30:1006-7. doi: 10.1093/bioinformatics/btt730
2. Qin Y, Grimm SA, Roberts JD, Chrysovergis K and Wade PA (2020) Alterations in promoter interaction landscape and transcriptional network underlying metabolic adaptation to diet. Nat Commun 11:962. doi: 10.1038/s41467-020-14796-x
3. Pinero J, Ramirez-Anguita JM, Sauch-Pitarch J, Ronzano F, Centeno E, Sanz F and Furlong LI (2020) The DisGeNET knowledge platform for disease genomics: 2019 update. Nucleic Acids Res 48:D845-D855. doi: 10.1093/nar/gkz1021
